# Automatic Detection of Age-Related Macular Degeneration Based on Deep Learning and Local Outlier Factor Algorithm

**DOI:** 10.3390/diagnostics12020532

**Published:** 2022-02-18

**Authors:** Tingting He, Qiaoer Zhou, Yuanwen Zou

**Affiliations:** College of Biomedical Engineering, Sichuan University, Chengdu 610065, China; 2019223010062@stu.scu.edu.cn (T.H.); zhou_qiaoer@outlook.com (Q.Z.)

**Keywords:** age-related macular degeneration, optical coherence tomography, deep learning, local outlier factor

## Abstract

Age-related macular degeneration (AMD) is a retinal disorder affecting the elderly, and society’s aging population means that the disease is becoming increasingly prevalent. The vision in patients with early AMD is usually unaffected or nearly normal but central vision may be weakened or even lost if timely treatment is not performed. Therefore, early diagnosis is particularly important to prevent the further exacerbation of AMD. This paper proposed a novel automatic detection method of AMD from optical coherence tomography (OCT) images based on deep learning and a local outlier factor (LOF) algorithm. A ResNet-50 model with L2-constrained softmax loss was retrained to extract features from OCT images and the LOF algorithm was used as the classifier. The proposed method was trained on the UCSD dataset and tested on both the UCSD dataset and Duke dataset, with an accuracy of 99.87% and 97.56%, respectively. Even though the model was only trained on the UCSD dataset, it obtained good detection accuracy when tested on another dataset. Comparison with other methods also indicates the efficiency of the proposed method in detecting AMD.

## 1. Introduction

Age-related macular degeneration (AMD) is the fourth most prevalent ocular disease resulting in vision loss in the macula [1]. The macula is located in the optical center of the human eye and is an important part of the retina. It is required for reading, driving, watching TV, and performing many other daily activities [2]. Of all cases of blindness worldwide, 8.7% are caused by AMD and the number of patients with AMD was estimated at around 196 million in 2020, predicted to rise to 288 million by 2040 [3].

AMD is broadly classified into non-exudative or dry AMD and exudative or wet AMD. The difference between dry and wet AMD is that dry AMD does not have any blood or serum leakage. Around 85% to 90% of AMD cases are dry [4]. Patients suffering from dry AMD have a significant anomaly known as drusen in the retinal pigment epithelium (RPE) layer. The formation of drusen leads to a thinning and drying out of the macula, which results in the loss of macular function. Although patients with dry AMD may still have a good central vision, they may have significant functional limitations, including limited night vision, vision fluctuations, and reading difficulties due to a limited area of central vision. Moreover, a certain percentage of dry AMD may develop into wet AMD as time goes by [5]. In wet AMD, patients may see dark spots in their central vision due to blood or fluid leakage under the macula. The main pathogenesis of wet AMD is choroidal neovascularization (CNV), which occurs under the retina and macula. This neovascularization may lead to macular swelling and a reversible loss of vision, or bleeding, which can be highly toxic to the overlying photoreceptors, sometimes even causing irreversible vision loss [6,7]. In wet AMD, vision loss may be rapid and progressive. Once CNV has developed in one eye, the other eye is in a high-risk state and requires periodic eye examination [8]. Therefore, regular screening of the retina is crucial for the diagnosis and treatment of AMD and the prevention of further deterioration.

Ophthalmologists can detect AMD-related lesions through a variety of methods with the continuous development of imaging technology [9,10,11,12]. Optical coherence tomography (OCT) uses the basic principle of a weak coherence interferometer to carry out high-resolution cross-sectional tomography of the internal microstructure of materials or biological systems [13]. It has been widely used in many fields and has great scientific research and application potential in biomedical, agricultural and industrial detection [14,15,16,17,18]. In recent years, OCT has become a major tool to diagnose AMD and monitor its progress [19]. It reflects the physical structure of the retina accurately and effectively in a non-contact and non-invasive way [20,21]. It can clearly describe the particular pathology relating to AMD, such as drusen, intra-retinal fluid (IRF), sub-retinal fluid (SRF), sub-retinal hyper-reflective material and RPE detachment [22]. 

The OCT images to be analyzed increase dramatically with a more widespread screening of the retina. The number of professional ophthalmologists is limited because training new professional doctors is a long process. It is often difficult for patients of AMD to receive timely diagnosis and treatment, especially in places where medical resources are insufficient. Therefore, a computer-aided diagnosis (CAD) system that can automatically detect AMD from a large number of retinal OCT images is urgently needed. In recent years, researchers have launched kinds of studies on AMD detection and classification based on retinal OCT images with the development and improvement of computer technology and image processing algorithms [23,24,25,26,27,28,29,30,31,32,33,34].

## 2. Related Work

Layer segmentation is crucial in many automatic analysis algorithms based on retinal OCT images. The position and thickness of each retinal layer are obtained according to the result of the layer segmentation algorithm, then by analyzing the similarities and differences between the layer index of the tested image and the reference image, a variety of issues, including lesion detection and positioning, can be addressed.

Farsiu et al. [23] introduced a semi-automatic segmentation of RPE, RPE drusen complex (RPEDC) and total retina (TR) boundaries. Then, volumes of TR, RPEDC and abnormal RPEDC of each subject were measured and compared with the normal thickness generated by control subjects to detect AMD. The area under the curve (AUC) of the receiver operating characteristic (ROC) for this classifier was 0.9900. 

Naz et al. [24] proposed an algorithm to detect the AMD-effected OCT scans by calculating the difference between the RPE layer and a second-order polynomial curve. The method was made time efficient by using an intensity-based threshold method for the RPE segmentation. A dataset with 25 AMD and 25 healthy images was used, and the study obtained an accurate detection of AMD with 96.00% accuracy. 

Arabi et al. [25] used the binary threshold method to extract the RPE layer, sampled the extracted layers and counted the number of white pixels in each sample. The mean value of the numbers of pixels was calculated and classified. They tested the approach on 16 images and obtained an accuracy of 75.00%. 

Thomas et al. [26] proposed an algorithm based on RPE layer detection and baseline estimation using statistical methods and randomization for the detection of AMD from retinal OCT images. The method was tested on a public dataset including 2130 images and achieved an overall accuracy of 96.66%. 

Sharif et al. [27] presented a method based on feature extraction and the support vector machine (SVM). First, the RPE layer was extracted by utilizing the graph theory dynamic programming technique, then a unique feature set consisting of features extracted from the difference signal of RPE and the inner segment outer segment layer of RPE was obtained. Finally, the SVM classifier was used to detect AMD-affected images from 950 OCT scans, and an accuracy of 95.00% was obtained.

Although the above methods based on layer segmentation obtained promising results, they are not suitable for large-scale AMD detection. The convolutional neural network (CNN), which emerged at the end of the 20th century, has significantly improved the ability to classify images [28].

Lee et al. [29] classified 52,690 normal and 48,312 AMD OCT images utilizing a modified version of the VGG-16 CNN model and obtained an overall accuracy of 93.40%. Serener et al. [30] compared two pre-trained CNN, namely AlexNet and ResNet-18, to automatically classify OCT images for dry and wet AMD diseases, respectively. In both cases, the ResNet-18 model outperformed the AlexNet model, and the AUC of the ResNet model for each AMD stage was 0.9400 and 0.9300, respectively. 

Thomas et al. [31,32] conducted a number of studies based on AMD detection using OCT images. In [31], a multiscale and multipath CNN with six convolutional layers was proposed and finally achieved an overall accuracy of 98.79% with the random forest (RF) classifier. Later, in [32], they introduced another novel multiscale CNN with seven convolutional layers to classify AMD and normal OCT images. The multiscale convolution layer enables a large number of local structures to be generated with various filter sizes. The proposed CNN network finally achieved an accuracy of 99.73% on the UCSD dataset.

Yoo et al. [33] utilized VGG-19 pre-trained with images from ImageNet as a feature extractor, and a multiclass RF classifier was operated to detect AMD images. The overall accuracy using OCT alone was 82.60% on a small dataset including both OCT and matched fundus images. Kadry et al. [34] extracted handcrafted features, such as the local binary pattern (LBP), the pyramid histogram of oriented gradients (PHOG), and the discrete wavelet transform (DWT) from the test images and concatenated them with the deep features of VGG-16. The proposed technique achieved an accuracy of up to 97.00% for OCT images with different binary classifiers.

In this study, we presented a novel method for the detection of AMD based on OCT images and showed it to be more effective than existing methods. The rest of the paper is structured as follows. The proposed methodology is given in Section 3, then the datasets used for the experiment and the parameters of the model are given in Section 4. The experimental results and discussion are shown in Section 5. The conclusion is given in Section 6.

## 3. Method

**Figure 1:**
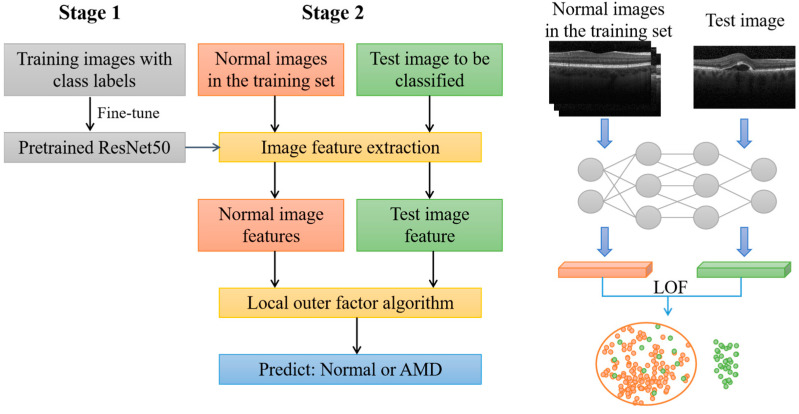
Framework of the proposed approach. Stage 1 is the process of training and Stage 2 is the process of feature extraction and classification. The diagram on the far right is a visual representation of Stage 2.

In this study, a two-stage model was proposed for the detection of AMD from OCT images, as shown in Figure 1. The first stage involved a classification model using a deep CNN, while in the second stage, an outlier detection method was used for detecting AMD.

In the first stage, a deep CNN based on ResNet-50 [35] was used for classification, and transfer learning was performed using AMD and normal OCT images [36]. After retraining, the last layer of the network (classification layer) was removed and the model was regarded as an image feature extractor. 

In the second stage, the normal images in the training set were imported to the network to obtain a normal image feature vector set. During testing, images in the test set were imported into the network in turn, and each test image could obtain a corresponding feature vector. Both the normal image feature vector set and test image feature vector were used as inputs of the local outlier factor (LOF) algorithm [37]. Finally, the LOF algorithm classified the test image as normal or abnormal (corresponding to AMD).

### 3.1. ResNet-50

ResNet was proposed by He et al. [35]. In the deep structure of CNN, as the layers deepen, gradient disappearance or explosion may occur, resulting in a drop in accuracy. The problem can be solved by the residual network, improving the performance and increasing the depth of the network at the same time. ResNet has been widely used in the field of medical image classification, in applications such as multi-label chest X-ray classification [38], the diagnosis of COVID-19 [39] and exudate detection in fundus images [40].

**Figure 2:**
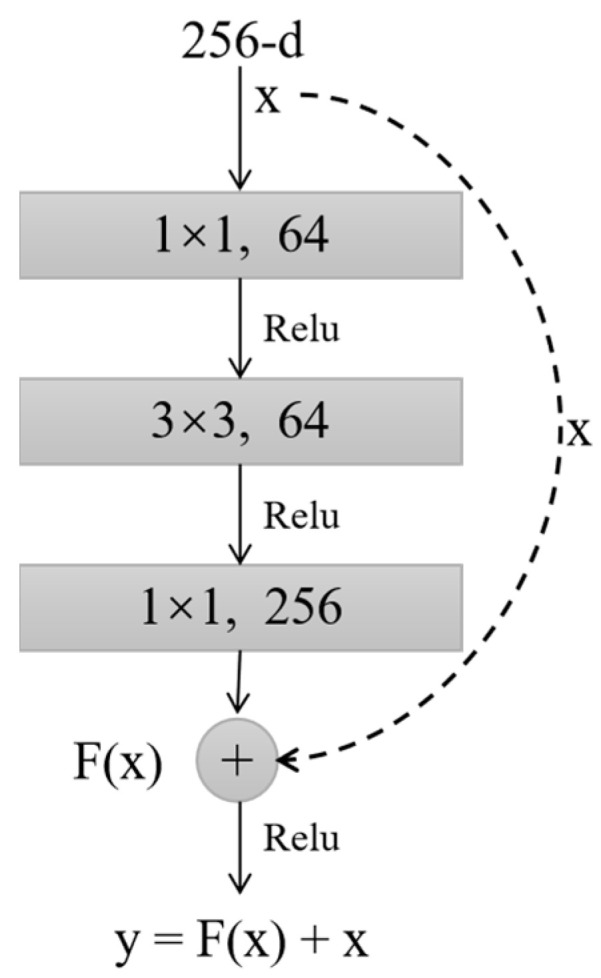
A “Bottleneck” building block for ResNet-50, where the 1 × 1 layers are responsible for reducing and then increasing (restoring) dimensions, leaving the 3 × 3 layer with a bottleneck of smaller input/output dimensions.

In this work, we utilized a 50-layer structure of ResNet. The residual block structure of ResNet-50 is shown in Figure 2. The input *x* is transferred across layers through a shortcut connection to be added into the output *F(x)* after convolution, and then the output *y =* *F(x) + x* is obtained. The residual block can fully train the underlying network, so the accuracy can be significantly improved as the depth increases.

**Figure 3:**
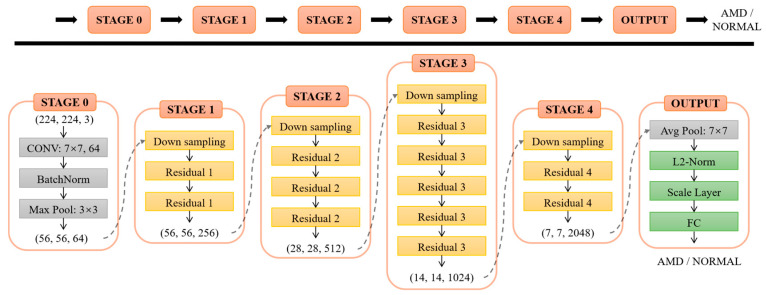
The architecture of the ResNet-50 used. L2-constrained is implemented via the L2-Norm layer and the Scale layer in the OUTPUT module.

Figure 3 shows the architecture of ResNet-50 used in this study. The last fully-connected layer was adjusted to binary output classes for AMD and normal instead of the 1000 output classes of the ImageNet, and the loss function was L2-constrained softmax loss [41].

The L2-constrained softmax loss is given by Equation (1) [41]:

(1) $$\begin{array}{l} minimize-\frac{1}{M}\sum_{i=1}^{M}log\frac{e^{W_{y_{i}}^{T}f(x_{i})+b_{y_{i}}}}{\sum_{j=1}^{C}e^{W_{y_{i}}^{T}f(x_{i})+b_{y_{i}}}}\\ subject\;\;to\;\;||f(x_{i})|{|}_{2}=\alpha ,\forall i=1,2,3\ldots M \end{array}$$

where *x_i_* is the input image in the mini-batch of size *M*, *y_i_* is the corresponding class label, *f(x_i_)* is the feature descriptor obtained from the penultimate layer, *C* is the number of classes, and *W* and *b* are the weight and bias of the last layer. An additional L2 constraint is added on the basis of the traditional softmax function, and the constraint is enforced by adding an L2-normalize (L2-Norm) layer, followed by a scale layer, as shown in Figure 3. This constraint restricts the feature to a hypersphere with a fixed radius through a parameter, which brings the features from the same class closer to each other and separates the features from different classes in the normalized or angular space. In this study, *M* = 12, *C* = 2, and *α* was set to 5. 

### 3.2. LOF Algorithm

The LOF algorithm was used to divide OCT images into two groups: normal and AMD. The LOF is an outlier detection method that computes the local density deviation of a given data point with respect to its neighbors [37]. The local density is given by Equation (2):

(2) $$lrd_{k}(p)=1/(\frac{\sum_{o\in N_{k}(p)}reach-dist_{k}(p,o)}{\left|N_{k}(p)\right|})$$

where *lrd_k_(p)* is the local density of object *p*, *N_k_(p)* is the k-distance neighborhood of *p*, and *reach-dist_k_(p,o)* is the reachability distance of object *p* with respect to object *o*, which is given by Equation (3):

(3) $$reach-dist_{k}(p,o)=max\left\{k-distance(o),d(p,o)\right\}$$

**Figure 4:**
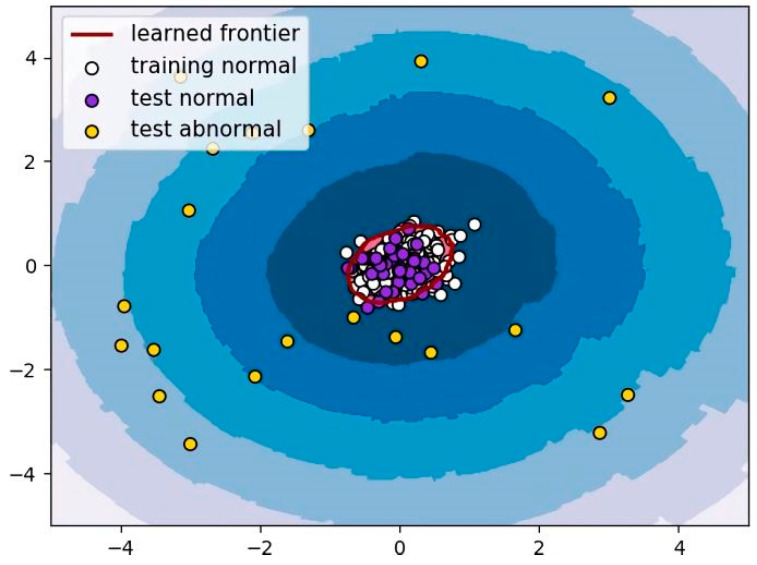
Schematic illustration of LOF. LOF can be used to determine the threshold of normal OCT images based on the local density of the normal images in the training set, and discriminate whether the test image is normal or abnormal (AMD).

Using *lrd_k_(p)*, the LOF of *p* is defined by Equation (4):

(4) $$LOF_{k}(p)=(\frac{\sum_{o\in N_{k}(p)}\frac{lrd_{k}(o)}{lrd_{k}(p)}}{\left|N_{k}(p)\right|})$$

where *k* was set to 20 in this study. The output *LOF* value was used to determine whether *p* was normal or abnormal (corresponding to AMD), as shown in Figure 4.

## 4. Experiment

This section consists mainly of two parts: datasets and model training, including parameters and the environment.

### 4.1. Datasets

The proposed method was trained and validated using the UCSD dataset [42]. The UCSD dataset was selected from retrospective cohorts of adult patients between 1 July 2013 and 1 March 2017, and all OCT images were acquired using Spectralis OCT (Heidelberg Engineering, Heidelberg, Germany) imaging. There were 45,821 AMD images in total, including 8616 dry AMD and 37,205 wet AMD, and 80% of the images were used as the training set and 20% of the images as the validation set, as given in Table 1.

The UCSD test set contains 500 AMD images (250 dry AMD and 250 wet AMD) and 250 normal images. In addition, we also tested the method with the Duke dataset [43]. The Duke dataset includes multiple OCT images from 45 subjects (15 dry AMD, 15 DME, and 15 normal) and all images were acquired in Institutional Review Board-approved protocols using Spectralis OCT (Heidelberg Engineering Inc., Heidelberg, Germany) imaging. The proposed method utilized images from AMD and normal subjects.

**Figure 5:**
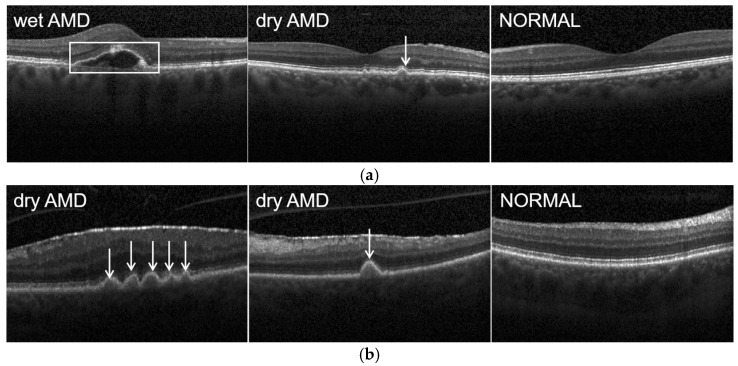
Sample images: (**a**) belongs to the UCSD dataset; (**b**) belongs to the Duke dataset. The white arrows point to drusens in the dry AMD, and the white box shows the irregular blood vessel under the macula in the wet AMD.

The sample images in the datasets are shown in Figure 5. Dry AMD is a state in which the macula layer becomes thin and dry. There is a small amount of amorphous material that aggregates in the cells of the eye, also known as drusen, as shown by the white arrows in Figure 5. Wet AMD refers to the irregular blood vessel under the macula, which is called CNV. This blood vessel may cause the macula to rise from its flat position due to fluid leakage and bleeding, as shown by the white box in Figure 5a.

### 4.2. Model Training

All images were resized to 224 × 224 × 3 and divided into mini-batches. During the process of retraining the ResNet-50, the Adam optimizer was used and the parameters of all layers in ResNet-50 were updated. The batch size was 12, the learning rate was 0.001 and the number of epochs was 30.

The study was implemented on a machine with an Intel (R) Xeon (R) CPU E5-2680 v4 @ 2.40 GHz processor and an NVIDIA Tesla P40 graphics card. The experiments were based on the open-source deep learning frameworks TensorFlow-gpu 2.1.0 and Keras 2.3.1. 

## 5. Results and Discussion

### 5.1. Performance Evaluation

The results of the proposed work are evaluated based on the confusion matrix. In the field of machine learning, the confusion matrix is a visual tool to measure the performance of classification. As shown in Table 2, each column of the matrix represents the label results predicted by the model, and each row of the matrix represents the real label of the sample.

In the confusion matrix, TP and TN give properly classified data results, while FP and FN give wrongly classified information. We can measure accuracy, precision, sensitivity, and F1-score using these values to analyze the performance of the proposed method.

Accuracy is the ratio of the number of samples classified correctly to the total number of OCT images, and is calculated using the following equation:

(5) $$\mathrm{Accuracy}=\frac{\mathrm{TP}+\mathrm{TN}}{\mathrm{TP}+\mathrm{TN}+\mathrm{FP}+\mathrm{FN}}$$

Sensitivity refers to the proportion of all AMD images that are correctly classified as AMD, measuring the ability of the classifier to detect AMD images, and is calculated using the following equation:

(6) $$\mathrm{Sensitivity}=\frac{\mathrm{TP}}{\mathrm{TP}+\mathrm{FN}}$$

Precision represents the proportion of all images predicted to be AMD that are actually AMD images, and is calculated using the following equation:

(7) $$\mathrm{Precision}=\frac{\mathrm{TP}}{\mathrm{TP}+\mathrm{FP}}$$

F1-score specifies the harmonic mean of precision and recall, and is calculated using the following equation:

(8) $$F1-\mathrm{score}=2\times \frac{\mathrm{Precision}\times \mathrm{Sensitivity}}{\mathrm{Precision}+\mathrm{Sensitivity}}$$

In addition, the ROC curve and the value of AUC are used to measure the overall classification performance of the proposed method.

### 5.2. Results

#### 5.2.1. UCSD Dataset 

**Figure 6:**
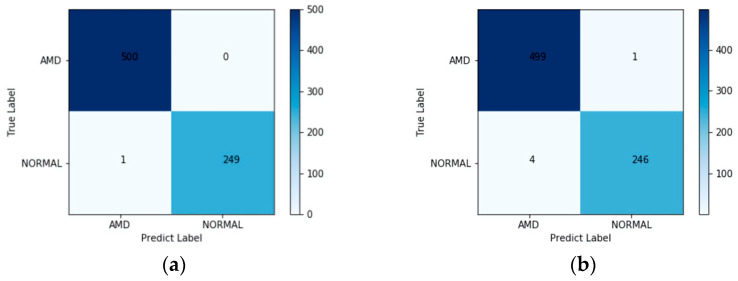

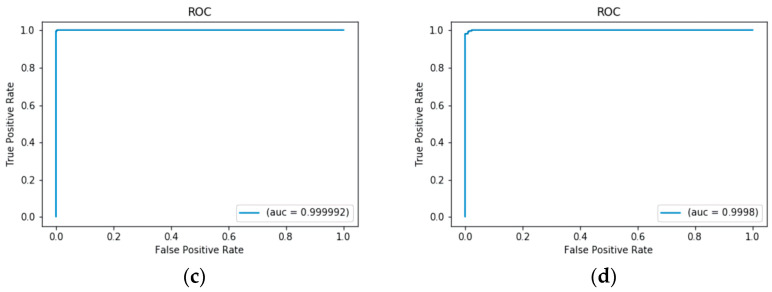
(**a**) Confusion matrix of the proposed method on the UCSD test set. (**b**) Confusion matrix of ResNet-50 on the UCSD test set. (**c**) ROC curve of the proposed method on the UCSD test set. (**d**) ROC curve of ResNet-50 on the UCSD test set.

The confusion matrix of the proposed method, tested on the UCSD test set, is shown in Figure 6a. The test set consisted of 250 dry AMD, 250 wet AMD, and 250 normal images, so we took a total of 500 AMD images and 250 normal images for testing. All of the AMD images were correctly classified, and in normal images, only 1 image out of 250 was mistakenly classified as AMD. The ROC curve of the proposed approach on the UCSD test set is plotted in Figure 6c and the value of AUC is very close to 1.0000. 

We also directly used the retrained ResNet-50 as a classifier to verify the effectiveness of the combination of deep learning and LOF algorithms. The confusion matrix and the ROC curve are shown in Figure 6b,d, respectively. In total, 1 out of 500 AMD images and 4 out of 250 normal images were classified mistakenly, and the value of the AUC was 0.9998. The results show that deep learning combined with the LOF algorithm can classify OCT images more efficiently.

The accuracy, sensitivity, precision, F1-score and AUC obtained on the UCSD test set are shown in Table 3. It is clear that the AMD possessed a better accuracy than normal. The weighted average evaluation parameters, such as F1-score, sensitivity, and precision, were greater than 99.80%, with an AUC value very close to 1.0000. Hence, it shows that the proposed method performs well in the classification of AMD and normal OCT images. 

A comparison of the proposed method with existing methods conducted on the UCSD dataset is given in Table 4. Thomas et al. [18,19] proposed two novel CNN architectures specifically for AMD and normal classification based on OCT images, obtaining an accuracy of 99.78% and 99.73%, with an AUC of 0.9978 and 0.9999, respectively. Other previous works obtained less than 98.50% accuracy and less than 0.9920 AUC. In the case of the UCSD dataset, the proposed method obtained a higher average accuracy and AUC than other methods and showed better performance in detecting AMD. 

#### 5.2.2. Duke Dataset

**Figure 7:**
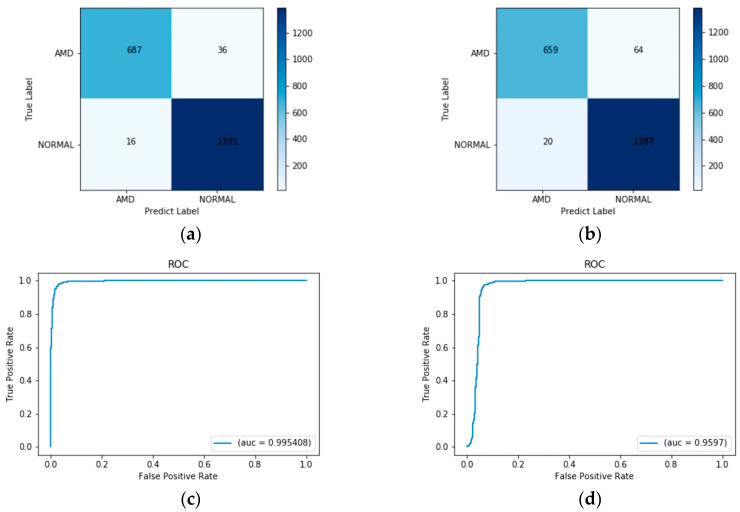
(**a**) Confusion matrix of the proposed method on the Duke test set. (**b**) Confusion matrix of ResNet-50 on the Duke test set. (**c**) ROC curve of the proposed method on the Duke test set. (**d**) ROC curve of ResNet-50 on the Duke test set.

The proposed method was trained only on the UCSD dataset but was tested on two publicly available datasets, the UCSD dataset and the Duke dataset. The Duke dataset consists of 1407 normal images, and 723 AMD images [30]. The confusion matrix of the Duke test set is given in Figure 7a. Out of 723 AMD images, 36 of them were misclassified as normal. In normal images, 16 images out of 1407 were misclassified as AMD. The ROC curve of the proposed work on the Duke test set is plotted in Figure 7c, and the value of AUC was approximately 0.9954. The confusion matrix and the ROC curve when testing with ResNet-50 on the Duke test set are also given in Figure 7b,d, respectively. It is obvious that the proposed method, combining ResNet-50 and the LOF algorithm, performed better than using ResNet-50 alone.

The performance evaluation of the Duke test set is tabulated in Table 5. The results of the proposed method tested on the Duke dataset are not as excellent as those in the UCSD dataset, but still obtained an overall weighted accuracy of 97.56%.

The comparison of the proposed method with existing methods conducted on the Duke dataset is given in Table 6. The approach proposed by Hussain et al. [35] obtained the best classification accuracy of 97.70%, with an AUC of 0.9900. First, they segmented the retina layers, then extracted features such as the thickness of the retina and the thickness of the individual retinal layers, and the volume of the pathologies such as drusen and hyper-reflective intra-retinal spots. Finally, the classification result was obtained using an RF classifier. The other previous works achieved an accuracy of less than 97.00%.

According to the above analysis, even if the proposed method is trained only on the UCSD dataset, it also achieved good detection accuracy when tested on the Duke dataset. Additionally, the proposed method was compared with the method using ResNet-50 alone, as shown in Figure 6 and Figure 7. The comparison was also made with previous methods, such as those in [26,31,32,42,43,44,45,46,47,48,49,50,51], as shown in Table 4 and Table 6, based on accuracy and AUC. The results suggest that the proposed method performed very well compared with previous models when classifying AMD and normal OCT images.

## 6. Conclusions

A novel automatic detection method was presented for the detection of AMD from OCT images based on deep learning and an outlier detection method. The ResNet-50 model with L2-constrained softmax loss was retrained to extract features from OCT images, and the LOF algorithm was used as the classifier. The proposed method was trained on the UCSD dataset and tested on both the UCSD dataset and the Duke dataset, with an accuracy of 99.87% and 97.56%, respectively. Even though the model was only trained on the UCSD dataset, it also performed well when tested on the Duke dataset. Table 4 and Table 6 show the comparison of the proposed method with existing works, which also indicates the efficiency of the proposed method in detecting AMD.

The advantage of the proposed method is its excellent ability to classify AMD and normal OCT images without preprocessing and with high accuracy, which will help doctors in large-scale OCT image screening.

Our proposed method achieves a good overall performance in the detection of AMD, enabling the proposed method to be used for the early detection of AMD. In future work, we hope to use more datasets to validate the proposed method, and based on this study, we will further subdivide the AMD into dry AMD and wet AMD.

## Figures and Tables

**Table 1:** Data distribution for training and validation using the UCSD dataset.

UCSD Dataset	AMD	NORMAL	TOTAL
Training set (80%)	36,656	40,912	77,568
Validation set (20%)	9165	10,228	19,393

**Table 2:** Binary-classification confusion matrix.

True Label	Predict Label	
	AMD	NORMAL
**AMD**	True Positive (TP)	False Negative (FN)
**NORMAL**	False Positive (FP)	True Negative (TN)

**Table 3:** Quantitative results of the proposed method on the UCSD test set.

Class	Accuracy %	Sensitivity %	Precision %	F1-Score %	AUC
AMD	100.00	100.00	99.80	99.90	-
NORMAL	99.60	99.60	100.00	99.80	-
Weighted Average	99.87	99.87	99.87	99.87	1.0000

**Table 4:** Comparison of the proposed method with existing works conducted on the UCSD dataset.

Method	Weighted Average Accuracy %	AUC
Multi-scale and multi-path CNN [31]	99.78	0.9978
Multi-scale CNN [32]	99.73	0.9999
Inception V3 transfer learning [42]	96.53	0.9762
Multi-scale deep feature fusion [44]	97.71	0.9900
AlexNet transfer learning [45]	98.26	0.9917
Iterative fusion CNN [46]	93.40	0.9798
Proposed method	**99.87**	**1.0000**

**Table 5:** Quantitative results on the Duke test set.

Class	Accuracy %	Sensitivity %	Precision %	F1-Score %	AUC
AMD	95.02	95.02	97.72	96.38	-
NORMAL	98.86	98.86	97.48	98.17	-
Weighted Average	97.56	97.56	97.56	97.56	0.9954

**Table 6:** Comparison of the proposed work with existing works conducted on the Duke dataset.

Method	Weighted Average Accuracy %	AUC
RPE detection and baseline estimation [26]	96.66	-
Feature extraction + SVM [43]	93.30	-
Intensity-based threshold + Ploy fitting curve [47]	92.00	-
Feature extraction + RF classifier [48]	**97.70**	0.9900
Feature extraction + Sequential Minimal Optimization [49]	96.60	0.9910
18-layer recombined residual CNN [50]	96.66	-
Sparse coding +Dictionary learning [51]	96.66	-
Proposed method	97.56	**0.9954**

## Data Availability

No new dataset was generated from this study. We utilized the following two public datasets in this study: https://data.mendeley.com/datasets/rscbjbr9sj/3 (accessed on 19 January 2022) and https://people.duke.edu/~sf59/Srinivasan_BOE_2014_dataset.htm (accessed on 19 January 2022).
